# Establishment and validation of a prognostic nomogram for extra-gastrointestinal stromal tumors

**DOI:** 10.3389/fonc.2025.1527237

**Published:** 2025-03-20

**Authors:** Guohua Jia, Xiangpan Li

**Affiliations:** Department of Head and Neck Oncology (including Lymphoma and Pediatric Oncology), Renmin Hospital of Wuhan University, Wuhan, China

**Keywords:** extra-gastrointestinal stromal tumors, nomogram, survival, prognostic model, risk factor

## Abstract

**Background:**

Extra-gastrointestinal stromal tumor (EGIST) is a rare subtype of gastrointestinal stromal tumor, with limited information on its clinical characteristics and prognostic factors. This study aims to identify the risk factors affecting survival in EGIST patients and to develop a prognostic nomogram for predicting EGIST-specific survival (TSS).

**Methods:**

Patients diagnosed with EGIST, aged 18 to 80, were enrolled from the Surveillance, Epidemiology, and End Results (SEER) Research Plus database, covering the years 2000 to 2019. Univariate and multivariate analyses were conducted to identify risk factors for developing a nomogram. The predictive accuracy of the nomogram was evaluated using time-dependent receiver operating characteristic curves, calibration plots, and the concordance index. Decision curve analysis (DCA) was performed to assess the nomogram’s clinical utility and net benefit for application in clinical practice. Additionally, the nomogram’s performance was compared with the tumor SEER stage.

**Results:**

A total of 389 patients were included in this study. Univariate and multivariate analyses identified age, household income, surgery, tumor grade, tumor size, and tumor SEER stage as significant predictors of TSS (all P<0.05). These factors were incorporated into the prognostic nomogram. The nomogram demonstrated superior prognostic value compared to the tumor SEER stage alone, as evidenced by the area under the curve and concordance index. The calibration plot indicated a high level of accuracy in forecasting survival probabilities. Furthermore, DCA highlighted the nomogram’s clinical applicability and positive net benefit. The nomogram also proved more effective than the tumor SEER stage alone in identifying patients with poorer prognoses.

**Conclusions:**

Age, household income, surgery, tumor grade, tumor size, and tumor SEER stage were identified as risk factors for TSS in patients with EGIST. We have developed and validated a prognostic nomogram to predict TSS in EGIST patients, which may improve patient management and guide personalized medical treatment for EGIST.

## 1 Introduction

Gastrointestinal stromal tumor (GIST) is the most common subtype of sarcoma, with an incidence rate between 1.1 and 1.5 per 100,000 persons (1, 2). GIST predominantly originates in the digestive tract, with the stomach accounting for 55% of cases, the small intestine for 31%, and the colorectum for 6%. Fewer than 5% of gastrointestinal stromal tumors occur outside the digestive tract and are classified as extra-gastrointestinal stromal tumors (EGIST) (2). Although EGIST shares similar pathological characteristics with GIST, it presents a distinct spectrum of clinical features. EGIST cases exhibit a higher proportion of poorly differentiated tumors, and a higher proportion of patients with lymph node or distant metastasis (3, 4). Additionally, the majority of GISTs are caused by activating mutations in KIT (75%–80%) or PDGFRA (5%–10%). However, the incidence of KIT or PDGFRA mutation is only 40–50% in EGIST patients, the lower mutation rate of KIT or PDGFRA in EGIST patients may influence the TKI treatment effort for EGISTs (5). As the incidence of EGIST is comparatively low, with most cases reported individually, the genomic research on EGIST is limited compared with GIST, there is a lack of whole-genome sequencing, whole-exome sequencing, whole-transcriptome sequencing, and circulating tumor DNA sequencing analyses, which have been proved to predict the prognosis of patients and guiding precision treatment for GIST patients (6, 7). Despite the existing knowledge gap concerning EGIST, there is currently no specific guideline tailored for EGIST. The current understanding of EGIST is scarce, and potential prognostic factors are poorly elucidated, leading to poor prognostic outcomes for EGIST patients. Despite limited knowledge regarding EGIST’s clinical aspects, these tumors remain a significant subset of GISTs, representing a unique entity with distinct characteristics and outcomes that merit further investigation (8–10). Therefore, identifying the risk factors associated with EGISTs and developing predictive models is essential for assessing patient prognosis and informing treatment decisions.

Nomograms are widely used prognostic tools, especially for predicting tumor prognosis, as they provide personalized risk assessments and support treatment decisions, enhancing clinical management’s precision of clinical management (11). Presently, EGIST’s prognostic implications are not well understood. While some nomograms predict the prognosis of GIST patients, they generally include only cases of GIST occurring within the gastrointestinal system or a small proportion of EGIST cases. Given the evidence highlighting the heterogeneity in clinical characteristics and prognosis between GIST and EGIST, these existing nomograms are inadequate for accurately predicting outcomes in EGIST patients with EGIST (12–14). Therefore, it is crucial to develop models that address EGIST’s unique characteristics to improve patient management and guide personalized treatment strategies.

This study aims to identify the risk factors influencing survival in EGIST patients and to develop and validate a novel nomogram specifically tailored for EGIST. The Surveillance, Epidemiology, and End Results (SEER) database, a valuable resource for cancer research, includes approximately half of all cancer cases in the United States (15). Data for this study were selected from the SEER database. Our nomogram integrates key demographic, clinicopathological, and therapeutic variables to construct a comprehensive tool for estimating patient survival probabilities. This nomogram aids in guiding treatment decisions and optimizing clinical management, aiming to deepen our understanding of EGIST and enhance clinical decision-making for its effective treatment.

## 2 Methods

### 2.1 Data sources

The data for this study were extracted from the SEER program (https://seer.cancer.gov), which provides comprehensive information from population-based cancer registries, including demographic data, tumor characteristics, antitumor therapy methods, and survival information. A data use agreement was executed to secure authorization for SEER database access. The dataset used for this investigation was SEER Research Data 17, submitted on November 12, and downloaded using SEER*Stat software (version 8.4.3, NY, USA).

### 2.2 Selection criteria

**Figure 1:**
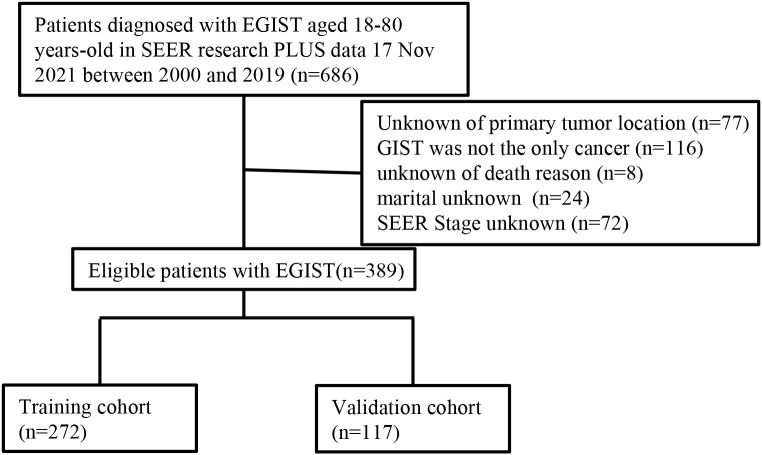
Flow diagram of the EGIST patients in the training and validation cohorts. EGIST: extra-gastrointestinal stromal tumors.

A total of 686 EGIST patients were identified based on the following criteria: (1) tumors histologically confirmed as GIST according to the ICD-O-3 recode; (2) patients aged between 18 and 80 years; and (3) primary tumor site outside the digestive tract, including locations such as the esophagus, stomach, small intestine, appendix, colon, and rectum. Exclusions included 77 cases with unknown primary tumor locations, 116 patients with multiple cancers, 8 cases missing cause of death, 24 cases with unknown marital status, and 72 patients lacking tumor SEER stage information. Ultimately, 389 patients remained for analysis. **Figure 1** illustrates the patient selection flowchart.

### 2.3 Parameter definition

Demographic information, disease characteristics, and treatment details for EGIST patients were obtained from the SEER database. Age was categorized as young adults (18-60 years) and older adults (>60 years). Race was classified as white, and others were classified based on race recode. Median household income, adjusted to 2019, was divided into low-income (≤USD 60,000), middle-income (USD 60,000–74,999), and high-income (≥USD 75,000) groups. The tumor SEER stage was categorized as follows: (1) localized stage for tumors confined to the site of origin; (2) regional stage for tumors with direct extension or regional lymph node metastasis; and (3) distant stage for metastasis to distant sites or lymph nodes. Vital status and cause of death were obtained from SEER data, with tumor-specific mortality (TSM) defined as deaths directly due to EGIST. Tumor-specific survival (TSS) was recorded months after diagnosis.

### 2.4 Construction of the nomogram

The eligible EGIST patients were randomly divided into a training cohort (n=272) and a validation cohort (n=117) in a 7:3 ratio. Univariate and multivariate Cox regression analyses were conducted to identify risk factors significantly affecting TSS in EGIST patients. Variables with statistical and clinical relevance were identified as independent risk factors and included in the nomogram development.

### 2.5 Evaluation of the nomogram

The nomogram’s performance was evaluated using time-dependent receiver operating characteristic (ROC) curves, the concordance index (C-index), calibration curves, and decision curve analysis (DCA). The ROC curve assessed the model’s sensitivity and specificity, with the area under the curve (AUC) indicating accuracy. The C-index represented predictive accuracy, while DCA evaluated the model’s net benefit. Calibration curves were used to compare predicted TSS with observed outcomes, with the 45-degree line as a reference for actual outcomes. The C-index and ROC were also employed to evaluate the nomogram’s prognostic accuracy compared to the tumor staging system.

### 2.6 Risk Classification of EGIST patients

Based on nomogram scores, EGIST patients were categorized into high-risk and low-risk groups, with the median nomogram score of the cohort (n=296) serving as the cutoff. Kaplan-Meier curve analysis compared survival differences across risk groups and tumor SEER stages.

### 2.7 Statistical analysis

All analyses were performed using R software version 4.3.1 (http://www.r-project.org/). The “survminer,” “regplot,” “mstate,” “survival,” “cmprsk,” “Hmisc,” “timeROC,” “foreign,” “nricens,” “rmda,” and “DCA” packages in R were used to develop and validate the nomogram. Statistical distribution differences between the training and validation cohorts were assessed using the chi-square test. All p-values were two-tailed, with p<0.05 considered statistically significant.

## 3 Results

### 3.1 Characteristics of patients

A total of 389 EGIST patients were included, randomized into a training cohort (n=272) and a validation cohort (n=117) in a 7:3 ratio. **Table 1** summarizes the demographic and clinical characteristics. Among the patients, 189 were young adults (48.59%), and 200 were older adults (51.41%). There were 201 male (51.67%) and 188 female patients (48.33%). The majority were white (67.87%, n=264). Tumor pathological grades were distributed as follows: grade I (7.71%), grade II (9.00%), grade III (5.91%), grade IV (7.46%), and unknown (69.72%). Regarding tumor size, 11.57% of patients had tumors <5 cm, 19.02% had tumors 5–10 cm, and 51.16% had tumors >10 cm. EGIST stage was localized in 34.19% of patients, regional in 25.96%, and distant in 39.85%. Surgical treatment was performed in 61.44% of patients, and 60.44% received chemotherapy. The marital status of 59.90% of patients were married. Low, middle, and high household incomes were observed in 24.94%, 43.44%, and 31.62% of patients, respectively. Comparison between the training and validation cohorts showed no significant differences in demographics and clinical characteristics (all P>0.05).

**Table 1:** Characteristics of patients with EGIST.

	Whole population, n (%)	training cohort, n (%)	validation cohort, n (%)	*p*
All	389 (100)	272 (100)	117 (100)	
Age				1
18-60	189 (48.59)	132 (48.53)	57 (48.72)	
>60	200 (51.41)	140 (51.47)	60 (51.28)	
Gender				0.119
Male	201 (51.67)	133 (48.90)	68 (58.12)	
Female	188 (48.33)	139 (51.10)	49 (41.88)	
Race				0.620
White	264 (67.87)	182 (66.91)	82 (70.09)	
Others	125 (32.13)	90 (33.09)	35 (29.91)	
Tumor Grade				0.650
I	30 (7.71)	21 (7.72)	9 (7.69)	
II	35 (9.00)	21 (7.72)	14 (11.97)	
III	23 (5.91)	18 (6.62)	5 (4.27)	
IV	29 (7.46)	20 (7.35)	9 (7.69)	
Unknown	272 (69.92)	192 (70.59)	80 (68.38)	
Tumor Size				0.260
≤5cm	45 (11.57)	33 (12.13)	12 (10.26)	
5-10cm	74 (19.02)	45 (16.54)	29 (24.79)	
>10cm	199 (51.16)	145 (53.31)	54 (46.15)	
Unknown	71 (18.25)	49 (18.01)	22 (18.80)	
Tumor SEER Stage				0.354
Localized	133 (34.19)	99 (36.40)	34 (29.06)	
Regional	101 (25.96)	67 (24.63)	34 (29.06)	
Distant	155 (39.85)	106 (38.97)	49 (41.88)	
Surgery				0.930
No	150 (38.56)	104 (38.24)	46 (39.32)	
Yes	239 (61.44)	168 (61.76)	71 (60.68)	
Chemotherapy				0.340
No	152 (39.07)	111 (40.81)	41 (35.04)	
Yes	237 (60.93)	161 (59.19)	76 (64.96)	
Marital Status				0.221
Single	156 (40.10)	115 (42.28)	41 (35.04)	
Married	233 (59.90)	157 (57.72)	76 (64.96)	
Household Income				0.557
Low	97 (24.94)	66 (24.26)	31 (26.50)	
Middle	169 (43.44)	123 (45.22)	46 (39.32)	
High	123 (31.62)	83 (30.51)	40 (34.18)	

EGIST, extra-gastrointestinal stromal tumors; SEER, Surveillance, Epidemiology, and End Result. Differences between the training and validation cohorts were assessed using the chi-square test.

### 3.2 Univariate and multivariate cox regression analyses

**Table 2** presents the univariate and multivariate Cox regression analyses Older adults (HR=1.59, 95%CI=1.08-2.34), tumor grade IV (HR=4.33, 95%CI=1.56-12.0), tumor size>10cm (HR=2.72, 95%CI=1.30-5.67), regional tumor (HR=2.38, 95%CI=1.33-4.28), distant tumor (HR=3.59, 95%CI=2.09-6.16) were identified as independent risk factors for TSS in EGIST patients; surgery treatment (HR=0.52, 95%CI=0.32-0.83), middle-household income (HR=0.54, 95%CI=0.35-0.85), and high-household income (HR=0.57, 95%CI=0.35-0.94) were independent protective factors for TSS in EGIST patients (all p<0.05).

**Table 2:** Univariate and multivariate Cox regression analyses of TSS in training cohort.

Variable	Univariate Analysis	*p*	Multivariate Analysis	*p*
	HR (95% CI)		HR (95% CI)	
Age				
18-60	Reference			
>60	1.66(1.15- 2.41)	0.007*	1.59(1.08-2.34)	0.020*
Gender				
Male	Reference			
Female	0.99(0.69-1.42)	0.943	1.05(0.71-1.55)	0.800
Race				
White	Reference			
Others	0.75(0.50-1.12)	0.162	0.78(0.51-1.21)	0.269
Tumor Grade				
I	reference			
II	0.92(0.28-3.00)	0.885	0.79(0.24-2.63)	0.703
III	2.39(0.85-6.74)	0.099	1.67(0.57-4.87)	0.35
IV	3.75(1.44-9.78)	0.007*	4.33(1.56-12.0)	0.005**
Unknown	1.93(0.84-4.43)	0.120	1.55(0.66-3.67)	0.316
Tumor Size				
≤5cm	Reference			
5-10cm	0.99(0.42-2.36)	0.989	1.14(0.47-2.76)	0.766
>10cm	2.07(1.03-4.15)	0.040*	2.72(1.30-5.67)	0.008**
Unknown	2.83(1.33-6.01)	0.007**	2.26(0.98-5.23)	0.056
Tumor SEER Stage				
Localized	Reference			
Regional	2.09(1.21-3.63)	0.009**	2.38(1.33-4.28)	0.0036**
Distant	4.34(2.66-7.06)	<0.001***	3.59(2.09-6.16)	<0.001***
Surgery				
No	Reference			
Yes	0.41(0.28-0.60)	<0.001***	0.52(0.32-0.83)	0.006*
Chemotherapy				
No	Reference			
Yes	1.32(0.91-1.93)	0.146	0.72(0.47-1.10)	0.132
Marital Status				
Single	Reference			
Married	0.87(0.60-1.25)	0.450	0.73(0.49-1.09)	0.126
Household Income				
Low	Reference			
Middle	0.53(0.35-0.82)	0.004**	0.54(0.35-0.85)	0.007**
High	0.50(0.31-0.81)	0.004**	0.57(0.35-0.94)	0.026*

TSS, Extra-gastrointestinal stromal tumor-specific survival; HR, Hazard Ratio; 95CI, 95% Confidence Interval; *indicated *p* value <0.05, **indicated *p* value <0.01, ***indicated *p* value<0.001.

### 3.3 Construction and validation of the nomogram

**Figure 2:**
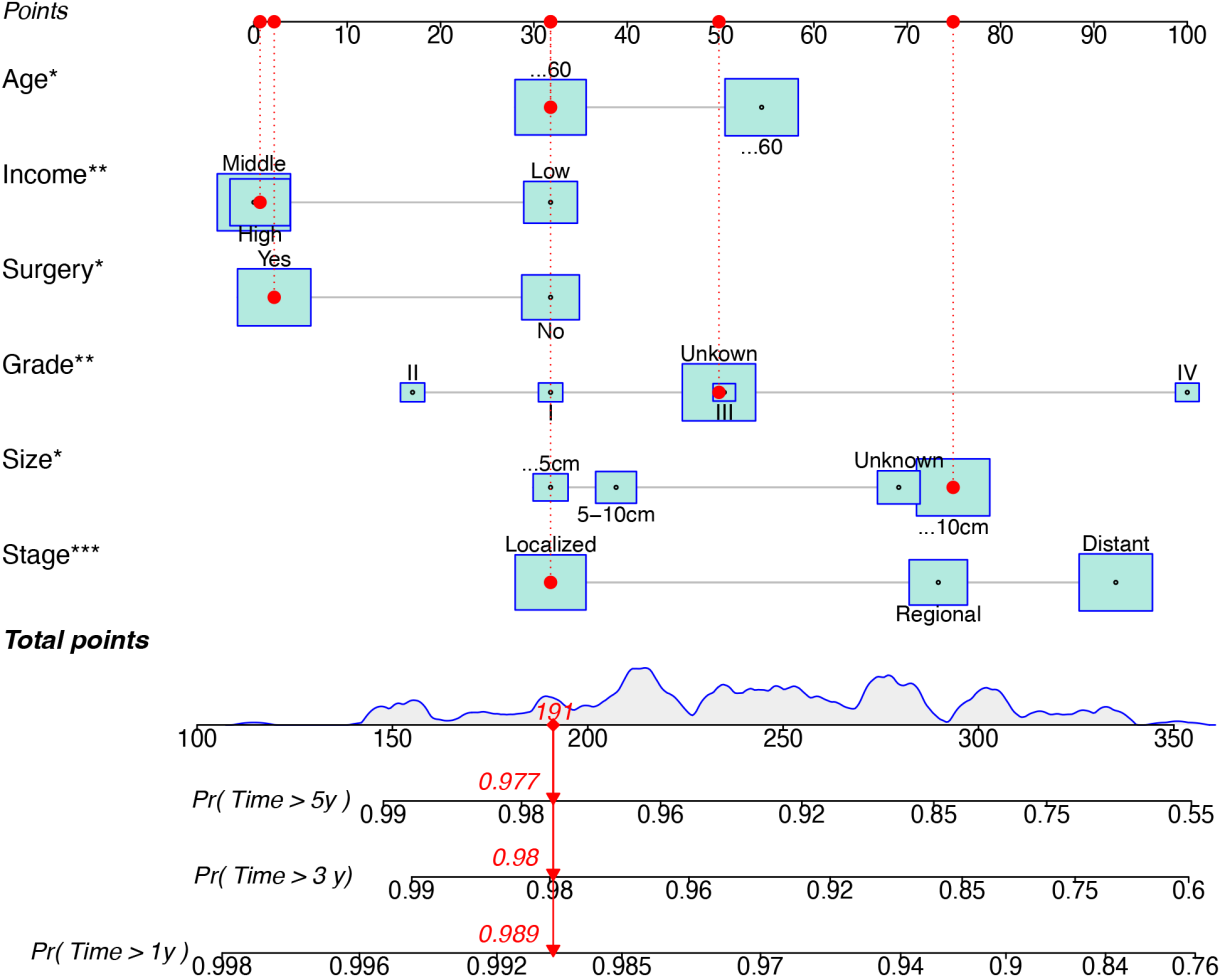
A nomogram for prediction TSS for EGIST patient. TSS, Extra-gastrointestinal stromal tumor-specific survival; EGIST, extra-gastrointestinal stromal tumors. *indicated p value < 0.05, **indicated p value < 0.01, and ***indicated p value < 0.001 in multivariate Cox regression analyses for predicting TSS for EGIST patients.

**Figure 3:**
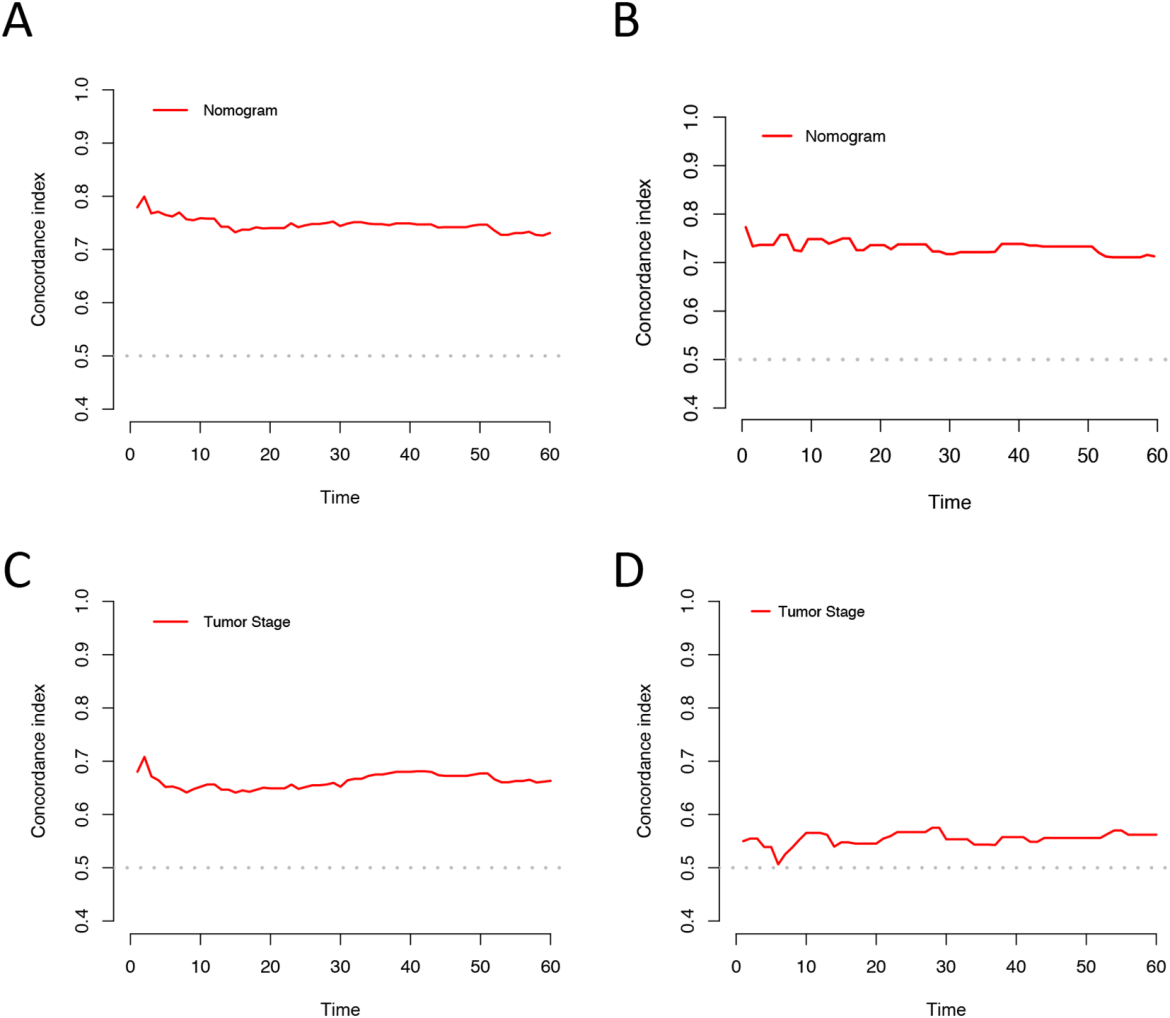
C-index analysis for the nomogram and tumor SEER stage prediction. **(A)** Training cohorts based on the nomogram. **(B)** Validation cohorts based on the nomogram. **(C)** Training cohorts based on the tumor SEER stage. **(D)** Validation cohorts based on the tumor SEER stage.

**Figure 4:**
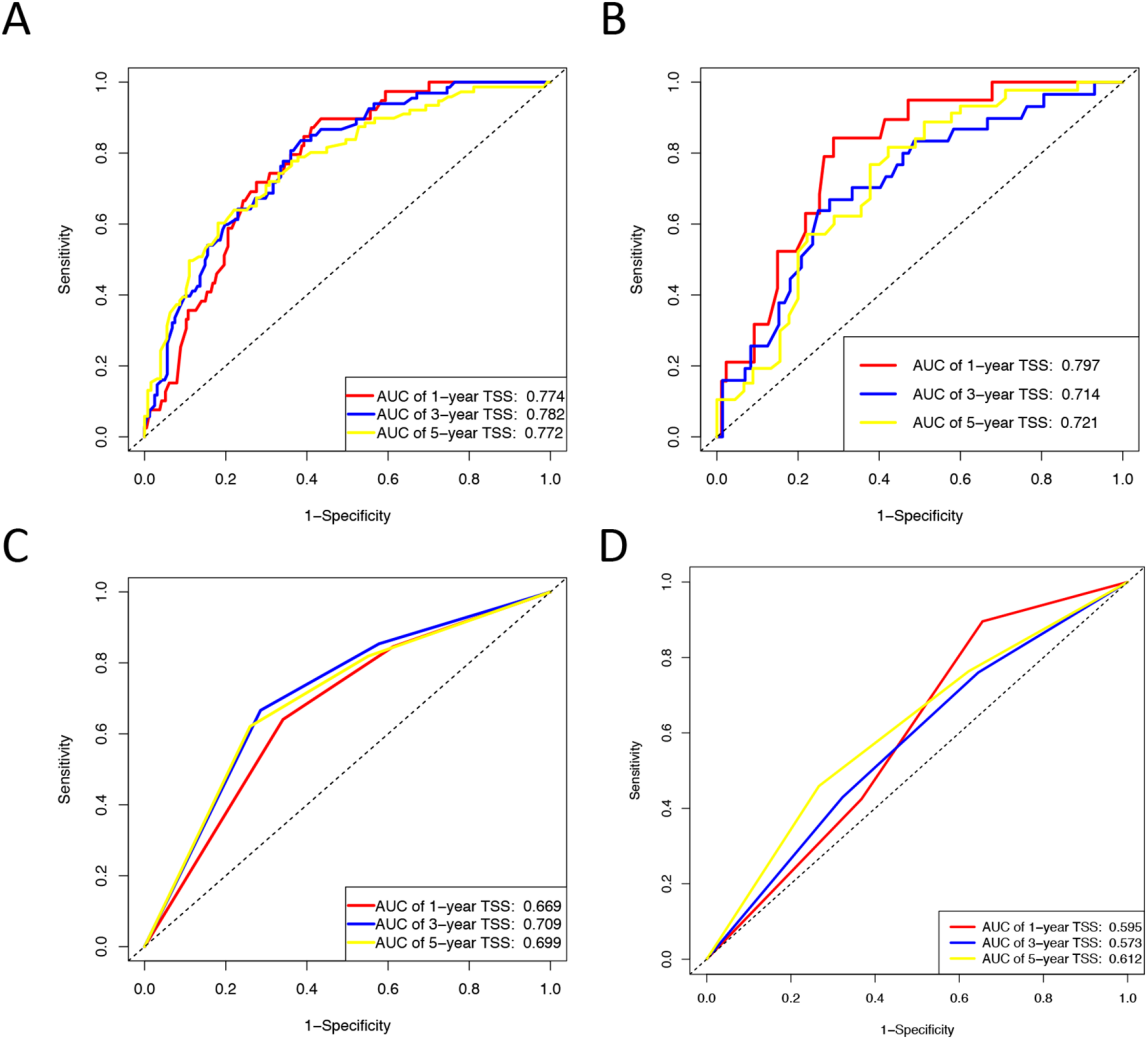
ROC curves for the nomogram and Tumor SEER Stage for 1-, 3-, and 5-year prediction. **(A)** Training cohorts based on the nomogram. **(B)** Validation cohorts based on the nomogram. **(C)** Training cohorts based on the tumor SEER stage. **(D)** Validation cohorts based on the tumor SEER stage.

**Figure 5:**
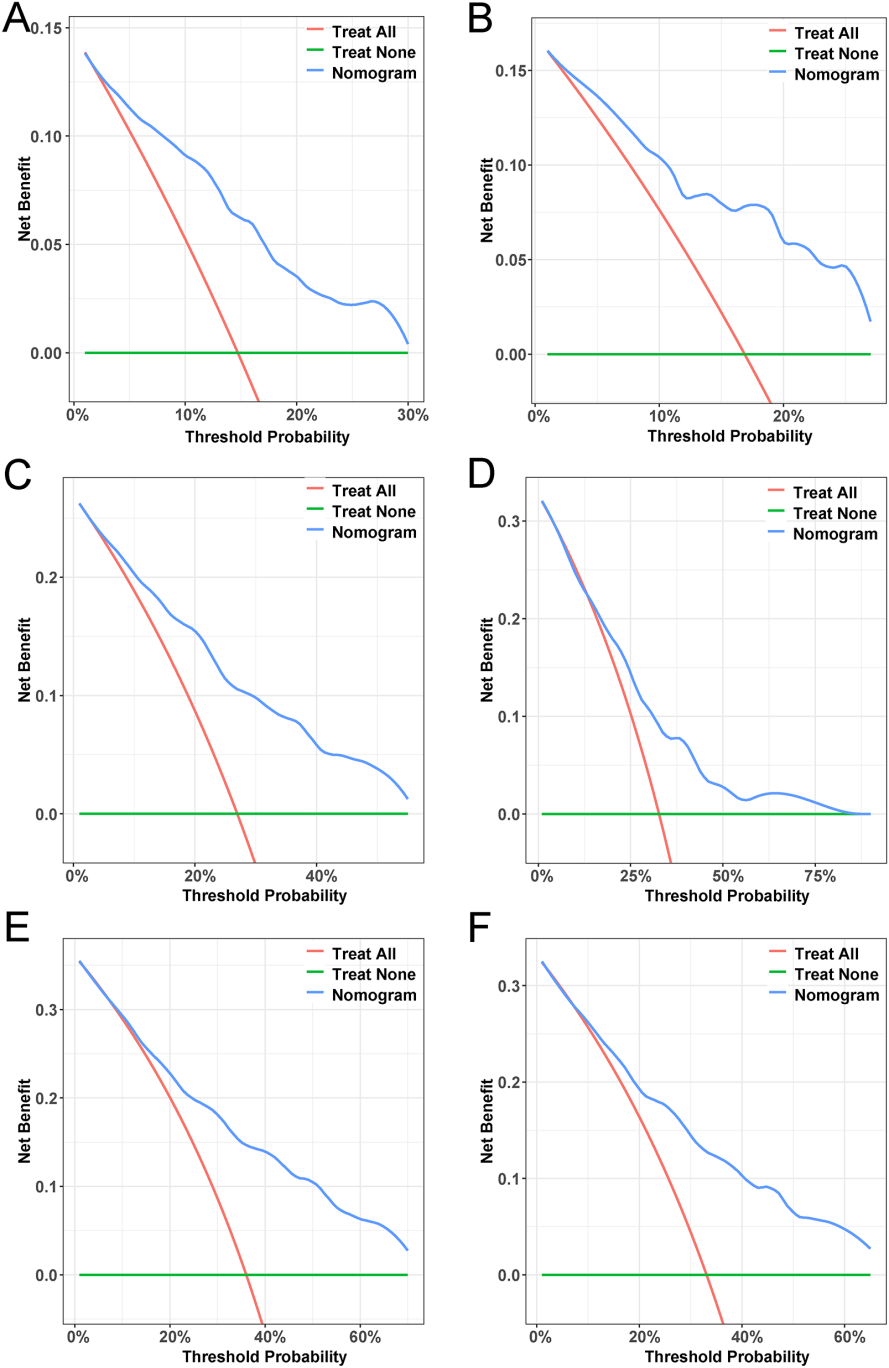
Decision curve analysis. **(A, C, E)** DCA curves of 1-year, 3-year, and 5-year TSS in the training cohort. **(B, D, F)** DCA curves of 1-year, 3-year, and 5-year TSS in the validation cohort. DCA, decision curve analysis; TSS, Extra-gastrointestinal stromal tumor-specific survival.

**Figure 6:**
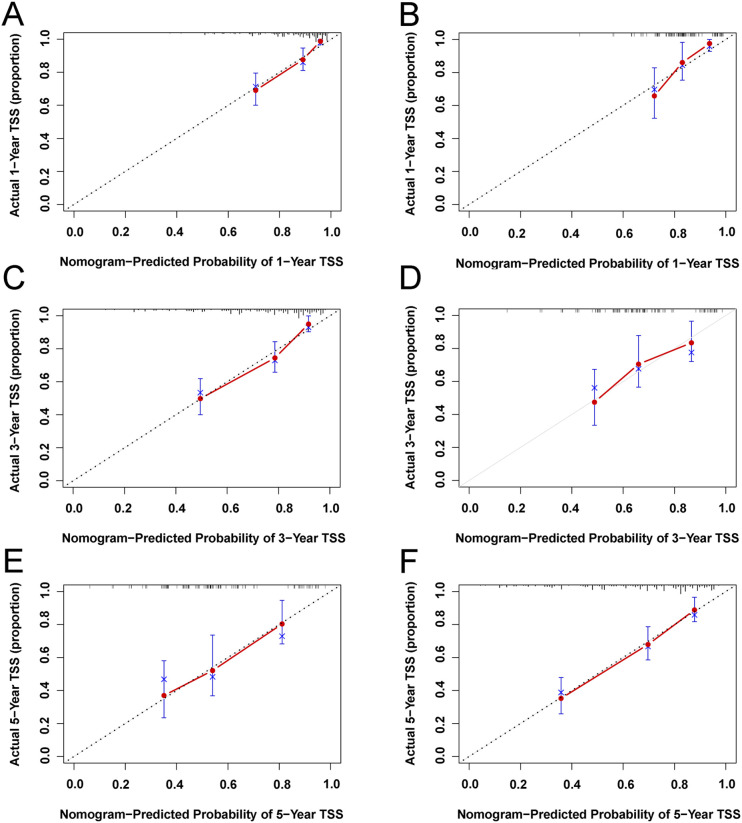
Calibration plots of 1-year, 3-year, and 5-year TSS for EGIST patients. **(A, C, E)** Calibration plots of 1-year, 3-year, and 5-year TSS in the training cohort. **(B, D, F)** Calibration plots of 1-year, 3-year, and 5-year CSS in the training cohort. TSS, Extra-gastrointestinal stromal tumor-specific survival.

Based on univariate and multivariate Cox regression analyses, age, tumor grade, tumor size, tumor SEER stage, surgery, and household income were selected to construct the nomogram for predicting TSS in EGIST patients (**Figure 2**). The risk scores for each variable were calculated and aggregated to determine total scores, with the probability of 1-, 3-, and 5-year TSS estimated by drawing a straight line on the final three rows. The C-index for the training and validation cohorts was 0.75 and 0.73, respectively (**Figures 3A, B**). The ROC curves showed AUC values of 0.77, 0.78, and 0.77 for 1-, 3-, and 5-year TSS in the training cohort, and 0.80, 0.71, and 0.72 in the validation cohort, indicating good predictive performance (**Figures 4A, B**). DCA curves showed good clinical applicability and positive net benefit in both cohorts (**Figure 5**). The calibration curves showed agreement between predicted and observed TSS rates at 1, 3, and 5 years (**Figure 6**).

### 3.4 Clinical value of the nomogram compared to tumor SEER stage based on SEER staging

The C-index and ROC were used to compare the accuracy of the nomogram and tumor staging system. The C-index of the nomogram was 0.75 and 0.73 in the training and validation cohorts, respectively, while the tumor SEER stage system had C-index values of 0.66 and 0.55 (**Figure 3**). The 1-, 3-, and 5-year ROC curves for the nomogram in the training cohort had AUC values of 0.77, 0.78, and 0.77, compared to 0.67, 0.71, and 0.70 for the staging system. In the validation cohort, the nomogram’s AUC values were 0.80, 0.71, and 0.72, whereas the staging system had AUC values of 0.60, 0.57, and 0.61 (**Figure 4**). The higher AUC and C-index values demonstrate that the nomogram provides superior TSS prediction accuracy compared to the tumor staging system.

### 3.5 Establishment of a stratified risk system based on the nomogram

**Figure 7:**
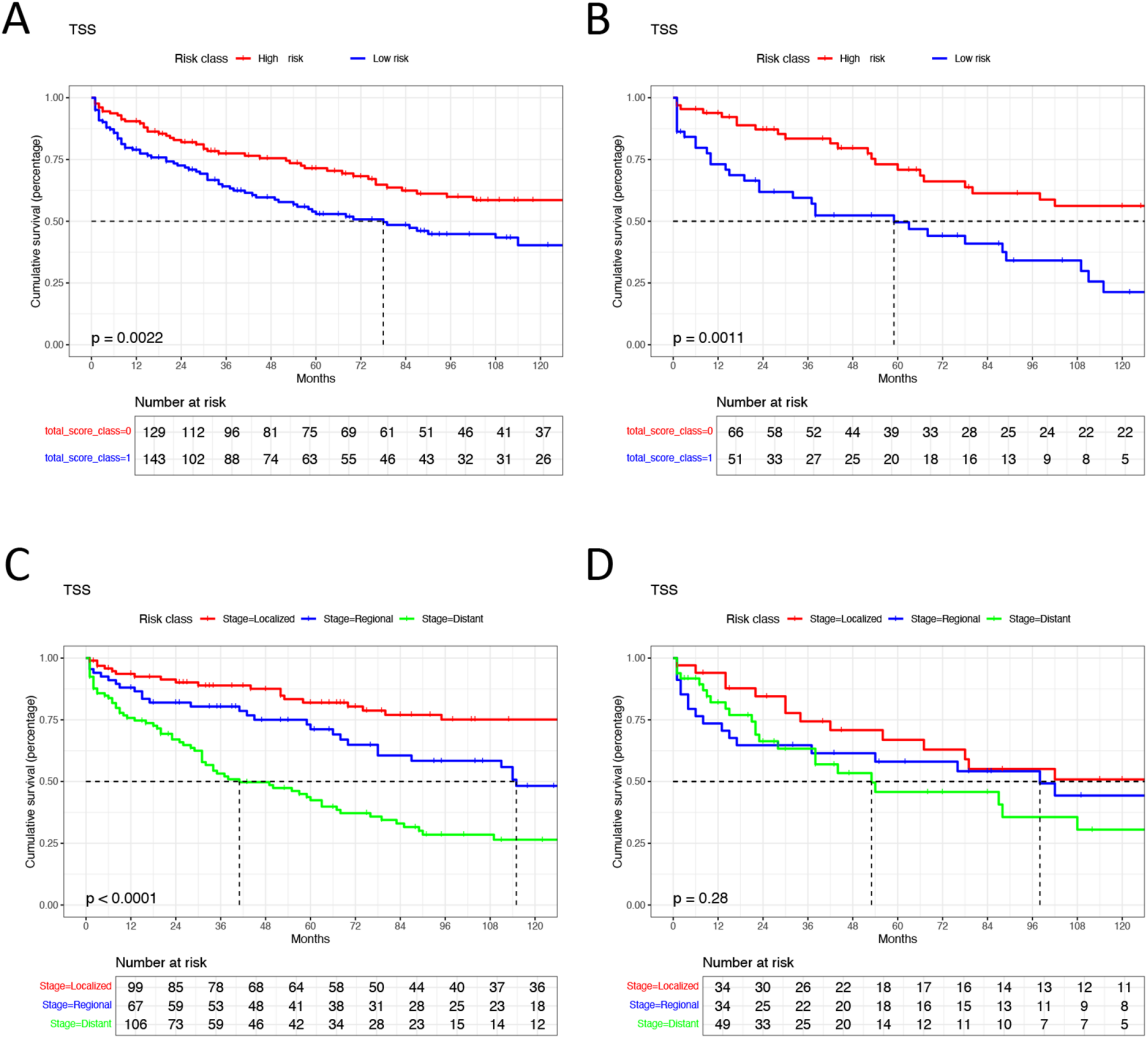
Kaplan-Meier analysis in different groups. **(A)** high risk group and low risk group in training cohorts. **(B)** high risk group and low risk group in validation cohorts. **(C)** different tumor SEER stage group in training cohorts. **(D)** different tumor SEER stage group in validation cohorts. TSS, Extra-gastrointestinal stromal tumor-specific survival.

Patients with EGIST were stratified into low-risk (total points <296) and high-risk (total points ≥296) groups according to the nomogram. Kaplan-Meier analysis showed a significant difference in TSS between the low-risk and high-risk groups. In contrast, the tumor staging system had limited capacity (63%) to differentiate patients with poor prognoses in training and validation cohorts (**Figure 7**).

## 4 Discussion

In this study, we identified patient age, tumor grade, tumor size, tumor SEER stage, surgical treatment, and household income as risk factors significantly associated with TSS in patients diagnosed with EGIST. Using data from the SEER database, we developed a nomogram to predict TSS for EGIST patients. This prognostic tool demonstrated robust performance in predicting patient prognosis and effectively-identified high-risk groups with poorer outcomes who may require closer monitoring and intervention. Our nomogram represents a step forward in understanding EGIST and supports the advancement of personalized medicine for patients with this rare tumor type.

EGIST, a rare form of GIST occurring outside the gastrointestinal tract, comprises approximately 5% of all GIST cases. While EGIST shares common c-kit and PDGFR-α mutations with GIST, it displays distinct clinical behaviors and prognostic characteristics (16). EGIST has received attention due to its aggressive nature and unique clinical outcomes compared to GIST within the gastrointestinal tract. Most existing studies on EGIST consist of case reports, with limited systematic analyses of clinical characteristics and prognostic factors. Using the SEER database, our study gathered a large cohort of EGIST patients, enabling us to systematically summarize clinical features and evaluate risk factors.

Our study identified several key sociodemographic characteristics among patients with EGIST. Socioeconomic factors, such as household income, have been shown to influence survival outcomes across various cancers (17). Our study further identified that low-household income is an independent risk factor for the survival of patients with EGIST, a finding that aligns with previous research on GIST (13). However, socioeconomic distributions varied significantly across different regions, given these disparities, it is essential to conduct cross-national comparative studies to explore the experiences and lessons learned by different countries and regions in addressing socioeconomic inequalities. Despite these variations, our study underscores the importance of public health strategies that target EGIST patients, particularly those with lower socioeconomic status. While gender has been identified as a prognostic factor for GIST survival, with evidence indicating that female GIST patients tend to have better survival outcomes than male patients, our research findings are consistent with prior EGIST studies, showing no significant difference in survival by gender (18–20). Further studies are needed to elucidate the detailed reasons. Furthermore, racial and marital status did not significantly affect EGIST survival, unlike GIST, where such factors have been influential (20, 21). The differences of sociodemographic characteristics between EGIST and GIST patients remain notable. The management of EGIST differs from that of GIST patients, which requires specific strategies. Developing personalized treatment plans and psychological support programs specifically designed for EGIST is crucial for improving the prognosis of these patients.

The oncologic characteristics of EGIST also differed from GIST. A clinical study comparing 62 EGIST cases with 570 GIST cases demonstrated that EGIST patients had a higher incidence of larger tumor sizes (>10 cm), more frequent signs of tumor necrosis, and higher mitotic rates, all of which correlate with poor prognosis (19). Our study also shows that 51.2% EGIST have tumor larger than 10cm, 44.4% were grade III-IV, 65.8% have a regional or distant tumor at diagnosis, which indicated EGIST have a higher degree of malignancy. EGIST’s origin remains controversial, with some researchers viewing it as a distinct subtype of GIST and others regarding it as a form of metastasis from the gastrointestinal tract (3, 5, 22). However, EGIST’s unique clinical features, prognostic factors, and outcomes support its classification as a separate subtype rather than a metastatic variant of GIST. Furthermore, our study excluded individuals with multiple tumors. The evidence of non-gastrointestinal origin further reinforces the notion that EGIST represents a distinct subtype of GIST. The higher degree of malignancy and poorer prognosis observed in EGIST underscore the limitations of current understanding of this tumor. To bridge the knowledge gap concerning EGIST, it is essential to explore its genomic characteristics and identify novel therapeutic biomarkers. Such efforts will enhance our comprehension of the pathogenesis underlying EGIST and ultimately contribute to improving its prognosis.

The treatment modalities for EGIST are typically based on GIST protocols, mainly include surgical resection, targeted therapy, and comprehensive treatment strategies. Surgery remains the primary treatment option for both GIST and EGIST (10, 23–25). However, high-risk EGIST patients often have poorer outcomes than GIST patients after surgery, indicating that adopting GIST-based strategies for EGIST may lead to suboptimal treatment (19). Radiotherapy has shown benefits for metastatic or progressive GIST (26). However, in two retrospective studies on EGIST, no patients received radiotherapy, and there was only one EGIST patient received radiotherapy in our study, making it difficult to evaluate its potential as a treatment option for EGIST (19, 27). More studies are needed to determine whether, and which subgroups of EGIST patients, will benefit from radiotherapy. For unresectable or metastatic EGIST, targeted therapies remain the preferred treatment option. However, when used as primary treatment, targeted therapies achieve a median overall survival of only 3.1 years for EGIST patients, compared to 6.9 years for GIST patients (27, 28). Given the absence of EGIST-specific treatment guidelines and large randomized controlled trials, treatment strategies for EGIST based on GIST protocols lead to poor outcome, further study should focus on the personalized and precision treatments for EGIST.

A nomogram designed for EGIST could address this need by predicting patient survival and categorizing risk levels, aiding treatment decisions, and potentially improving outcomes.

In GIST research, several nomograms have been developed to predict survival outcomes, incorporating age, race, tumor location, mitotic rate, chemotherapy, tumor size, stage, grade, and more (29–31). Sun et al. (32) created a nomogram for GIST patients post-surgery, while Bertsimas et al. (33, 34) developed prognostic models to predict the recurrence of GIST and assist in selecting patient groups suitable for adjuvant imatinib therapy. However, since patients with EGIST exhibit distinct prognostic risk factors compared to those with GIST, the existing nomograms are not applicable to EGIST patients. Our study used prognostic factors including age, tumor grade, size, tumor SEER stage, surgery, and household income to develop a nomogram predicting TSS in EGIST patients. The ROC and C-index values demonstrated the nomogram’s accuracy, and calibration curves indicated high concordance with observed outcomes. DCA further supported the nomogram’s clinical applicability with a positive net benefit. To our knowledge, our study involved the largest cohort of EGIST patients and introduced the first nomogram explicitly designed for this tumor type. Additionally, we stratified patients into high- and low-risk groups using the nomogram, with the high-risk group showing significantly poorer TSS outcomes. While the SEER staging system was limited in identifying patients with poor prognoses, our risk stratification approach effectively differentiated these patients, potentially guiding personalized and targeted treatment.

Our study has several strengths. It included the largest known cohort of EGIST patients, allowing for a comprehensive evaluation of clinical characteristics and risk factors. Additionally, we restricted the study to patients with a single primary tumor located outside the gastrointestinal tract, reducing the potential for misclassification of EGIST as metastatic GIST. There are also some limitations in our study, the SEER database spans multiple decades, and data gaps exist for some variables. Additionally, some patients were missing key characteristics such as tumor grade and tumor size. Moreover, given the extremely low incidence rate of EGIST, it is hard to collect sufficient external validation data. Therefore, we used internal validation data to assess the reliability and reproducibility of the nomogram. Despite these limitations, the high AUC and C-index values indicate that our nomogram is accurate and practical, supporting its use in predicting prognosis and guiding treatment.

## 5 Conclusion

We identified age, household income, surgical treatment, tumor grade, tumor size, and tumor SEER stage as significant risk factors for TSS in EGIST patients. Our prognostic nomogram offers a valuable tool for predicting TSS, enhancing patient management, and guiding personalized medical treatment for EGIST patients.

## Data Availability

Publicly available datasets were analyzed in this study. This data can be found here: The data used in this study can be accessed through the SEER program (https://seer.cancer.gov/).
